# Simultaneous Intake of Euglena Gracilis and Vegetables Synergistically Exerts an Anti-Inflammatory Effect and Attenuates Visceral Fat Accumulation by Affecting Gut Microbiota in Mice

**DOI:** 10.3390/nu10101417

**Published:** 2018-10-03

**Authors:** Yuto Sakanoi, Shuang E, Kazushi Yamamoto, Toshikuni Ota, Kentarou Seki, Mayumi Imai, Ryuki Ota, Yuta Asayama, Ayaka Nakashima, Kengo Suzuki, Tsuyoshi Tsuduki

**Affiliations:** 1 Laboratory of Food and Biomolecular Science, Graduate School of Agriculture, Tohoku University, Sendai 980-0845, Japan; s-noi.nod@ezweb.ne.jp (Y.S.); eshuang0@yahoo.co.jp (S.E); kazushi.yamamoto.t1@dc.tohoku.ac.jp (K.Y.); 2 Takeda Consumer Healthcare Company Limited, Chiyoda-ku, Tokyo 100-0005, Japan; toshikuni.oota@takeda.com (T.O.); kentarou.seki@takeda.com (K.S.); mayumi.imai@takeda.com (M.I.); ryuki.ota@takeda.com (R.O.); 3 Euglena Co., Ltd., Minato-ku Tokyo 108-0014, Japan; asayama@euglena.jp (Y.A.); nakashima@euglena.jp (A.N.); suzuki@euglena.jp (K.S.)

**Keywords:** *Euglena gracilis*, gut microbiota, inflammation, paramylon, vegetable, visceral fat

## Abstract

We determined whether the benefits provided by the consumption of *Euglena gracilis* (Euglena), which is a unicellular photosynthesizing green alga and rich in insoluble dietary fiber paramylon, can be enhanced by the co-consumption of vegetables that are rich in soluble dietary fiber. Nine-week-old male C57BL/6J mice were divided into four groups: group 1 received normal diet, whereas groups 2, 3 and 4 received normal diet containing 0.3% paramylon, 1.0% Euglena, or 1.0% Euglena plus 0.3% vegetables (barley leaf, kale and ashitaba), respectively. Mice were fed ad libitum until 18 weeks of age. Euglena intake significantly decreased serum markers of inflammation and co-consumption of vegetables enhanced this reduction. Notably, we observed an increase in the fraction of beneficial bacteria producing short-chain fatty acids, a reduction in harmful bacteria that cause inflammation and an increase in short-chain fatty acid production. Visceral fat accumulation was also reduced. Subsequent analyses showed that co-consumption of Euglena with vegetables reduced adipocyte area, suppressed the expression of genes related to fatty acid synthesis and increased the expression of genes related to adipocyte growth and lipolysis. Therefore, co-consumption of Euglena with vegetables enhanced its anti-inflammatory effect and the inhibitory effect on visceral fat accumulation likely by modulating the composition of gut microbiota.

## 1. Introduction

*Euglena gracilis* (Euglena) is a unicellular photosynthesizing green alga. It is rich in vitamins and minerals as well as in many other nutrients and it has recently attracted attention as a new health food [1]. Paramylon is an insoluble dietary fiber unique to Euglena with a triple-helical polymer structure composed of straight-chain β-1,3-glucans. Paramylon is known to attenuate manifestations of obesity and diabetes [2,3]. Paramylon also exhibits immunostimulatory activity by affecting cytokine production [4] and suppresses symptoms of atopic dermatitis and other chronic inflammatory disorders [5]. For these reasons, Euglena and paramylon have been the focus of many studies, although almost none of those revealed these beneficial effects in an efficient manner. It is therefore very important that effective methods of Euglena consumption are determined.

First, we focused on the properties of paramylon as a dietary fiber. Dietary fibers are broadly classified into soluble and insoluble dietary fibers [6]. Pectin, gum and storage polysaccharides are soluble dietary fibers, whereas cellulose, hemicellulose and lignin belong to insoluble dietary fibers [7]. Soluble dietary fiber interferes with the absorption of glucose and lipids from the intestinal tract, which reduces the concentration of sugars and lipids in blood [8]. Soluble dietary fiber promotes the production of short-chain fatty acids from fermentation by intestinal bacteria, which enhances the defensive function of the intestines because short-chain fatty acids are used as an energy source by colonic mucosal bacteria [9]. Due to its tendency to absorb water, increase stool volume and improve bowel movement, insoluble dietary fiber also prevents colon cancer by helping the body to get rid of harmful substances [10]. Paramylon and almost all other dietary fibers found in Euglena belong to insoluble dietary fibers. To obtain an optimal combination of dietary fibers, we decided to use vegetables with high soluble dietary fiber content. The soluble dietary fibers contained in vegetables such as barley leaf, kale and ashitaba have been reported to delay the speed of absorption of excess nutrients from the intestines, promote the production of short-chain fatty acids by intestinal fermentation and improve intestinal environment [11]. In addition, the intake of Euglena and vegetables is able to be expected not only to strengthen dietary fiber but also beneficial effects by micronutrients and phytochemicals. This study investigated whether combining Euglena with vegetables was an effective method of maintain health by enhancing the benefits of Euglena. The investigation was carried out in mice and measured body composition, serological and liver parameters. We also analyzed gut microbiota, performed histological examination of white adipose tissue and measured expression levels of genes related to lipid metabolism. In addition, in order to verify the utility of intake of Euglena and vegetables as an effective way to maintain health, normal mice were raised on the test diet that based on a normal diet.

## 2. Materials and Methods 

### 2.1. Test Diets

In this study, the following test diets were prepared to examine in mice whether the effect of Euglena can be enhanced by its combination with vegetables: normal diet + paramylon, normal diet + Euglena (spray-dried powder of *Euglena gracilis*) and normal diet + Euglena + vegetables (mixture of spray-dried powder of *Euglena gracilis*, barley leaf (*Hordeum vulgare*), kale (*Brassica oleracea* var. *acephala*) and ashitaba (*Angelica keiskei*)). Diet-enriching samples were provided by Takeda Consumer Healthcare Company Limited and euglena Co., Ltd. (Tokyo, Japan). Paramylon is an indigestible dietary fiber, which is a major physiologically active substance of Euglena [1]. Vegetables that were combined with Euglena included barley leaf, kale and ashitaba, which all contain large amounts of water-soluble dietary fiber [11]. Paramylon, Euglena, or Euglena + vegetables samples were mixed with normal diet (98121701M, Research Diet Inc., New Brunswick, NJ, USA) (Table 1). Four test diets were prepared as follows: control diet—normal diet; paramylon diet—normal diet containing 0.3% paramylon (as Euglena contains 30% paramylon [1,2,3,4,5], 0.3% paramylon content functionally corresponded to 1% of whole Euglena content); Euglena diet—normal diet containing 1.0% Euglena; and Euglena + vegetables diet—normal diet containing 1.0% Euglena and 0.3% vegetables. The composition of each test diet is shown in Table 1. The energy content per 100 g of each test diet was 388 or 389 kcal. Energy was calculated using the modified Atwater method (4 kcal of protein, 9 kcal of lipid, 4 kcal of carbohydrate per g) [12,13].

### 2.2. Animals

All animal procedures were performed in accordance with the Animal Experiment Guidelines of the Tohoku University and the animal protocol was approved by the Animal Use Committee at the Tohoku University (2016AgA-009) [14,15]. Male C57BL/6J mice (9 weeks old) were obtained from Clea Japan (Tokyo, Japan). The mice had access to their respective diet and distilled water ad libitum in a temperature- and humidity-controlled room with a 12/12-h light/dark cycle. After acclimatization to the normal diet for 1 week, 40 mice were randomly divided into four groups. Each group consisted of 10 mice and they were divided into two cages. Mice were fed control diet, paramylon diet, Euglena diet, or Euglena + vegetables diet (CO, paramylon, Euglena, or Euglena + V groups, respectively) for 8 weeks. At 18 weeks of age, after a 12-h fast, the mice were weighed and blood samples were collected after decapitation. To obtain serum, the blood was centrifuged (900× *g*, 5 °C, 15 min). The brain, cecum, heart, lung, liver, spleen, pancreas, kidney and white adipose tissues were removed and weighed. Serum and organs were stored at −80 °C until use.

### 2.3. Serum and Liver Biochemical Analyses 

Biochemical analyses of serum and liver samples were performed as described previously [16,17]. Serum and liver triacylglycerol (TG), total cholesterol (TC) and serum phospholipid (PL), glucose, alanine aminotransferase (ALT) and aspartate transaminase (AST) levels were measured using commercial enzyme kits (Wako Pure Chemical, Osaka, Japan). Serum insulin and leptin were determined using an ELISA kit (Morinaga Institute of Biological Science, Yokohama, Japan). Liver phospholipids were measured using the procedure of Rouser et al. [18]. Serum IL-1β and IL-6 were determined using an ELISA kit (BD Biosciences, Franklin Lakes, NJ, USA). Serum and liver thiobarbituric acid reactive substances (TBARS) were measured as described previously [19]. A microplate reader (Infinite F200, Tecan Japan Co., Ltd., Kanagawa, Japan) was used for absorbance measurements.

### 2.4. Analysis of Gut Microbiota

DNA of intestinal bacteria was extracted from mouse stool samples and the gene encoding 16S rRNA was amplified by PCR. The composition of intestinal bacteria in feces was examined by meta 16S analysis as described previously [12,20]. Mouse feces were collected every day during the last week of the study period, DNA was extracted from all recovered feces and pooled for each group to make a stool sample. DNA extraction from mouse feces was carried out using a QIAmp DNA Stool Mini Kit (Qiagen, Hilden, Germany). PCR-based 16S rRNA gene amplicon sequencing was performed using 10 ng DNA, 10 µM of each barcoded forward and reverse primer, 2× Gflex PCR Buffer (Mg^2+^, dNTP plus) (Takara Bio, Otsu, Japan) and Tks Gflex DNA Polymerase (Takara Bio) in a total volume of 25 µL. To target 16S rRNA variable regions 3 and 4 (V3 and V4), a forward primer 341F (5-TCG TCG GCA GCG TCA GAT GTG TAT AAG AGA CAG CCT ACG GGN GGC WGC AG-3) and reverse primer 806R (5-GTC TCG TGG GCT CGG AGA TGT GTA TAA GAG ACA GGG ACT ACH VGG GTW TCT AAT-3) were used at 94 °C for 1 min followed by 28 cycles of 98 °C for 10 s, 50 °C for 15 s and 68 °C for 15 s. PCR products were tagged using a Nextera XT Index Kit (Illumina, San Diego, CA, USA) to distinguish sample IDs using 10 ng DNA, Nextera XT Index Primer1, Nextera XT Index Primer2, 2× Gflex PCR Buffer (Mg^2+^, dNTP plus) and Tks Gflex DNA Polymerase (Takara Bio, Otsu, Japan) in a total volume of 25 µL at 94 °C for 1 min followed by 8 cycles of 98 °C for 10 s, 60 °C for 15 s and 68 °C for 15 s. The final PCR products were mixed to give 2 ng of DNA in each sample. This final mixture was sent to Takara Bio for metagenomic analysis.

### 2.5. Short-Chain Fatty Acids (SCFAs) and Gamma-Aminobutyric Acid (GABA) Analyses 

SCFAs were derivatized and analyzed by liquid chromatography-tandem mass spectrometry (LC-MS/MS) (AB SCIEX, Framingham, USA). Acetic, propionic and butyric acid were quantified and the results were expressed in μmol/g of wet weight cecum content or μmol/L of serum. The details of the derivatization procedure and LC-MS/MS analysis of SCFAs were as described by Zeng et al. [21]. Serum GABA was determined using an ELISA kit (ImmuSmol SAS, Pessac, France) as described previously [16].

### 2.6. Histological Analysis

For histological analysis, epididymal adipose tissue was fixed in 10% formalin and embedded in paraffin [22,23]. Vertical sections (5 μm) were cut, mounted on a glass slide, stained with hematoxylin and eosin (HE) and observed using a microscope (BZ-9000; Keyence, Osaka, Japan). The mean area of adipocytes was calculated in each group [22,23].

### 2.7. mRNA Expression Analysis

For real-time quantitative reverse transcriptase PCR (qRT-PCR), total RNA was isolated from epididymal adipose tissue using an RNeasy Mini Kit (Qiagen, Valencia, CA, USA) [24], eluted with 40 μL of RNase-free water and stored at −80 °C until use. To quantify gene expression, mRNA levels for fatty acid synthase (*Fans*), glucose-6-phosphate dehydrogenase X-linked (*G6pdx*), malic enzyme (*Me*), peroxisome proliferator activated receptor gamma *(Pparg*), sterol regulatory element binding factor 1 (*Srebf1*) and -actin (*Actb*) in epididymal adipose tissue were determined with a Thermal Cycler Dice Real Time System^®^ (Takara Bio, Otsu, Japan). This system allows real-time quantitative detection of PCR products by measuring the increase in fluorescence caused by binding of SYBR green to double-stranded DNA [25]. In brief, cDNA was made using a Prime Script^®^ RT Master Mix (Perfect Real Time; Takara Bio, Otsu, Japan) from total RNA in epididymal adipose tissue. The cDNA was subjected to PCR amplification using SYBR^®^ Premix Ex TaqTM (Perfect Real Time; Takara Bio) and gene-specific primers for *Fas*, *G6pdx*, *Me*, *Pparg*, *Srebf1* and *Actb* (Table 2). PCR amplification was performed with activation at 95 °C for 10 s, followed by 40 cycles at 95 °C for 5 s (denaturation) and 60 °C for 31 s (extension) and dissociation at 95 °C for 15 s, 60 °C for 30 s and 95 °C for 15 s for each gene. Melting curve analysis was performed following each reaction to confirm the presence of only a single reaction product. The threshold cycle (Ct) represents the PCR cycle at which an increase in reporter fluorescence above a baseline signal can first be detected. The levels of mRNAs of genes of interest were normalized to that of *Actb* in test samples.

### 2.8. Statistical Analysis

Results are expressed as the mean ± standard error of the mean (SE). Data were analyzed by the one-way analysis of variance followed by the Tukey-Kramer post hoc test, if appropriate. All analyses were performed with a significance level of *α* = 0.05 (*p* < 0.05) using BellCurve for Excel (Social Survey Research Information Co., Ltd., Tokyo, Japan).

## 3. Results

### 3.1. Growth Parameters 

Body weights, food intake and tissue weights of experimental animals are shown in Table 3. There were no significant differences in body weights, food intake, or energy consumption between the groups. In addition, there were no significant differences in brain, heart, lung, liver, pancreas, spleen, kidney, or mesenteric adipose tissue weights between the groups. However, the weights of perinephric, epididymal and total white adipose tissue significantly decreased in Euglena and Euglena + V groups compared to those of CO group that fed the control diet, with Euglena + V group having the lowest white adipose tissue weight (Euglena + V vs. CO: *p* < 0.001, 0.001 and 0.001 for perinephric, epididymal and total white adipose tissues, respectively; Euglena vs. CO: *p* < 0.001, 0.005 and 0.005, respectively). Although white adipose tissue weights showed nominally lower values following the intake of paramylon, the effect of diet did not reach statistical significance in those cases. These results show that visceral fat accumulation was suppressed by ingesting Euglena and that simultaneous intake of Euglena and vegetables had a particularly strong effect.

### 3.2. Biochemical Parameters in Serum and Liver

**Figure 1:**
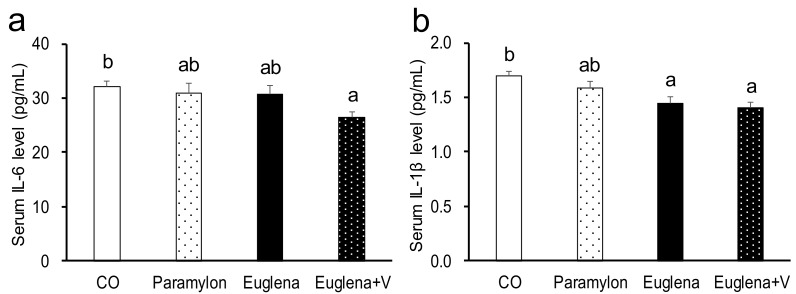
Effects of intake of Euglena and vegetables on inflammation parameters in mice. (**a**) Serum IL-6 level and (**b**) Serum IL-1β level are presented as the mean ± standard error of the mean, *n* = 10. Different superscript letters indicate significantly different means at *p* < 0.05 (refer to “Result” in detail). CO, control diet group; Paramylon, the group that received the normal diet containing 0.3% paramylon; Euglena, the group that received the normal diet containing 1.0% Euglena; Euglena + V, the group that received the normal diet containing 1.0% Euglena and 0.3% vegetables.

Biochemical parameters in serum and liver are shown in Figure 1 and Table 4. Serum IL-6, an index of inflammation, was significantly decreased in Euglena +V group compared to that in CO group (Euglena + V vs. CO, *p* < 0.05). Serum IL-1β, another index of inflammation, was also significantly lower in Euglena and Euglena +V groups compared to that in CO group, with Euglena + V group having the lowest IL-1β level (Euglena + V vs. CO, *p* < 0.05; Euglena vs. CO, *p* < 0.05, respectively). Liver TBARS, an index of lipid peroxidation, was significantly lower in Euglena +V group compared to that in CO group (Euglena + V vs. CO, *p* < 0.05). Although these parameters showed nominally lower values following the intake of Euglena or paramylon, the effect of diet did not reach statistical significance in those cases. Therefore, we concluded that inflammation was suppressed by the simultaneous intake of Euglena and vegetables.

### 3.3. Gut Microbiota

The above results showed that simultaneous intake of Euglena and vegetables had a greater impact than consumption of Euglena only. Thus, the beneficial effects appeared to be enhanced by vegetables. To clarify the factors involved in the enhancing effects of vegetables, changes in gut microbiota in Euglena and Euglena + V groups were examined. The 16S rRNA region of DNA extracted from feces was amplified and sequence analysis was performed using the next generation sequencer. Homology searches and lineage classification analyzes were performed on the 16S rRNA database using the obtained sequences and the genus levels of the microorganisms in the samples were determined. Detailed analysis of gut microbiota identified nine taxonomic units whose proportion was at least twice higher or lower in Euglena + V group compared with that in Euglena group (Table 5). *Erysipelotrichaceae; Other*, *YS2;f__;g__* and *Lactobacillus* were twofold more abundant in Euglena + V group compared with their levels in Euglena group. Furthermore, *Staphylococcus*, *Jeotgalicoccus*, *Sporosarcina*, *Streptococcus*, *Sutterella* and *Clostridiaceae; Other* were one-half or even less frequent in Euglena + V group compared with their levels in the Euglena group. It was not possible to determine the family and the genus of *YS2;f__;g__*. These differences in gut microbiota composition caused by the simultaneous intake of Euglena and vegetables likely were one of the factors underlying different effects of diets on physiological functions.

### 3.4. SCFAs and GABA Levels in Feces and Serum

**Figure 2:**
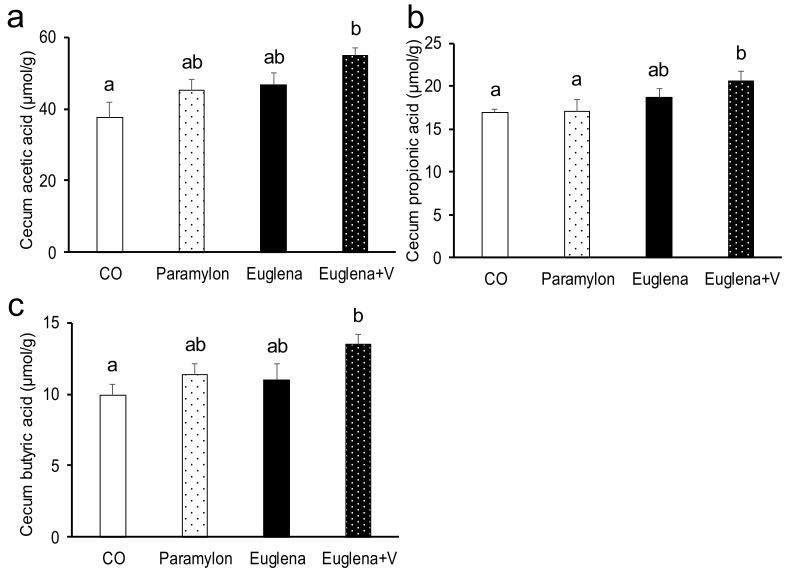
Effects of intake of Euglena and vegetables on SCFAs levels in cecum content of mice. (**a**) Cecum acetic acid level, (**b**) Cecum propionic acid level and (**c**) Cecum butyric acid level are presented as the mean ± standard error of the mean, *n* = 10. Different superscript letters indicate significantly different means at *p* < 0.05 (refer to “Result” in detail). CO, control diet group; Paramylon, the group that received the normal diet containing 0.3% paramylon; Euglena, the group that received the normal diet containing 1.0% Euglena; Euglena + V, the group that received the normal diet containing 1.0% Euglena and 0.3% vegetables.

**Figure 3:**
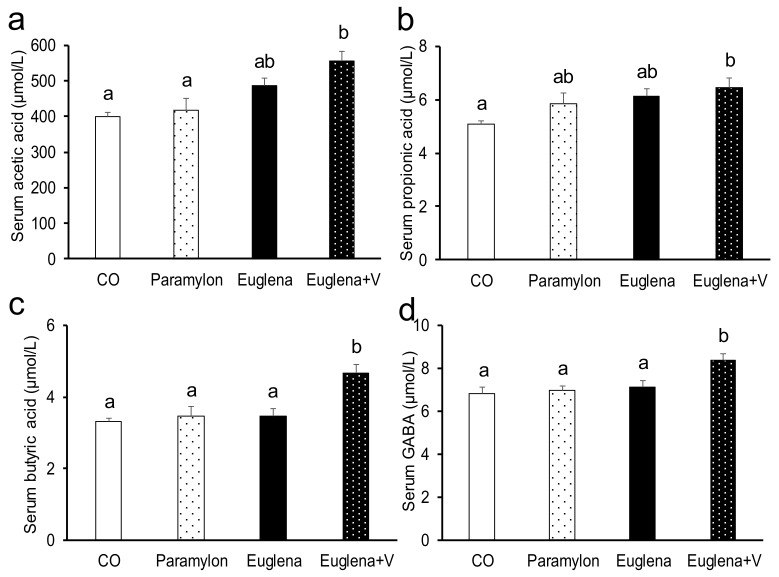
Effects of intake of Euglena and vegetables on SCFAs and GABA levels in serum of mice. (**a**) Serum acetic acid level, (**b**) Serum propionic acid level, (**c**) Serum butyric acid level and (**d**) Serum GABA level are presented as the mean ± standard error of the mean, *n* = 10. Different superscript letters indicate significantly different means at *p* < 0.05 (refer to “Result” in detail). CO, control diet group; Paramylon, the group that received the normal diet containing 0.3% paramylon; Euglena, the group that received the normal diet containing 1.0% Euglena; Euglena + V, the group that received the normal diet containing 1.0% Euglena and 0.3% vegetables.

Given that gut microbiota substantially changed due to simultaneous intake of Euglena and vegetables, the levels of SCFAs and GABA, whose production is promoted by intestinal bacteria, were measured (Figure 2 and Figure 3). Acetic, propionic and butyric acids levels in cecum content were significantly increased in Euglena + V group compared with the levels in CO group (Euglena + V vs. CO, *p* < 0.01, 0.01 and 0.05, respectively). Although SCFAs levels showed nominally higher values following the intake of Euglena or paramylon, the effect of diet did not reach statistical significance in those cases.

Acetic, propionic and butyric acids levels in serum significantly increased in the Euglena + V group compared with those in CO group (Euglena + V vs. CO; *p* < 0.05, 0.05 and 0.01, respectively). Although SCFAs levels showed nominally higher values following the intake of Euglena or paramylon, the effect of diet did not reach statistical significance in those cases. GABA level in serum was significantly higher in Euglena + V group than in CO group (Euglena + V vs. CO; *p* < 0.01). These results showed that the production of SCFAs and GABA was promoted by the simultaneous intake of Euglena and vegetables.

### 3.5. Histological Analysis of Epididymal Adipose Tissue

**Figure 4:**
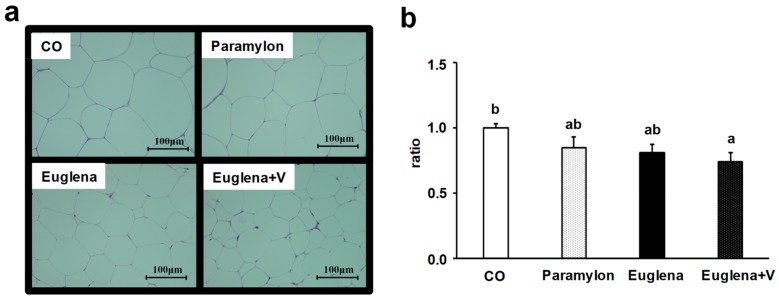
Effects of intake of Euglena and vegetables on white adipose tissue in mice. (**a**) Epididymal adipose tissue sections from representative mice in each group (hematoxylin & eosin, scale bar = 100 µm). (**b**) Adipocyte size ratio values are presented as the mean ± standard error of the mean, *n* = 10. Different superscript letters indicate significantly different means at *p* < 0.05 (refer to “Result” in detail). CO, control diet group; Paramylon, the group that received the normal diet containing 0.3% paramylon; Euglena, the group that received the normal diet containing 1.0% Euglena; Euglena + V, the group that received the normal diet containing 1.0% Euglena and 0.3% vegetables.

The large changes in adipose tissue weight suggested that the groups might differ in adipocyte size. Histological observations of epididymal adipose tissue, using HE staining (Figure 4a), showed that in Euglena and Euglena + V groups, adipocytes were smaller than in CO group. Quantification of adipocyte area showed a significantly lower value in Euglena + V group compared to that in CO group (*p* < 0.05) (Figure 4b). Although adipocyte area showed nominally smaller values following the intake of Euglena or paramylon, the effect of diet did not reach statistical significance in those cases. These results show that the expansion of adipocytes area was suppressed by Euglena consumption, with a particularly strong effect of the simultaneous intake of Euglena and vegetables.

### 3.6. mRNA Levels of Lipid Metabolism-Related Genes in Epididymal Adipose Tissue

The apparent suppression of visceral fat accumulation in the Euglena + V group prompted us to examine mRNA levels for lipid metabolism-related genes in epididymal adipose tissue that play a central role in visceral fat accumulation (Table 6). mRNA levels of *G6pdx* (fatty acid synthesis pathway enzyme) was lower in Euglena + V groups compared to that in CO group, which suggests that simultaneous intake of Euglena and vegetables suppressed the fatty acid synthesis system in the adipose tissue (Euglena + V vs. CO; *p* < 0.01). mRNA level of *Me* (fatty acid synthesis pathway enzyme) and *Srebf1* (transcription factor for these genes) were lower in Euglena + V group compared to those in paramylon group (Euglena + V vs. paramylon, *p* < 0.05 and 0.05, respectively). Euglena + V group had the lowest mRNA levels for these genes, showing that simultaneous intake of Euglena and vegetables suppressed the fatty acid synthesis system in the adipose tissue. mRNA levels for *Pparg*, which promotes the cell division and *Hsl*, which promotes lipolysis, increased in Euglena + V group compared to that in CO group (*p* < 0.05 in both cases), which suggests that simultaneous intake of Euglena and vegetables promoted cell division and lipolysis in the adipose tissue. There were no significant differences in *Fasn* mRNA level between the groups.

## 4. Discussion 

The objective of this study was to determine whether it is possible to enhance the benefits provided by consumption of Euglena, which is rich in insoluble dietary fiber, by consuming it with vegetables that are rich in soluble dietary fiber. Euglena intake caused a significant reduction in serum inflammatory markers in mice and this reduction was enhanced by co-ingestion of Euglena with vegetables. In the search for the mechanism of these diet-induced changes, we examined the effect of Euglena and vegetable co-ingestion on gut microbiota and found an increase in beneficial bacteria that produce short-chain fatty acids, a reduction in harmful bacteria that cause inflammation and an increase in the actual amount of short-chain fatty acids produced. We also observed a reduction in visceral fat in mice and this reduction was enhanced by co-ingestion of Euglena with vegetables. Histological examination of white adipose tissue and analysis of the expression of genes related to lipid metabolism revealed that co-ingestion of Euglena with vegetables caused a reduction in the area of adipocytes, suppressed the expression of genes related to fatty acid synthesis and increased the expression of genes related to adipocyte growth and lipolysis. Based on these findings, we propose that co-ingestion of Euglena with vegetables potentiates anti-inflammatory effect and enhances its suppressive effect on visceral fat accumulation. In addition, these results suggest that changes in gut microbiota are one of the mechanisms involved in these effects.

Although there were no significant differences in final body weight, mice that consumed paramylon tended to have a lower final body weight compared to the mice in CO group. Because the same result was also observed in mice that consumed Euglena only, this phenomenon presumably was due to paramylon presence. White adipose tissue weight also generally correlated with final body weight, with particularly large inter-group changes being observed around the kidneys and testicles. In a study of a type 2 diabetes model, visceral fat accumulation in rats that ingested paramylon or Euglena was suppressed and the effect was more prominent in rats that ingested Euglena compared to those that consumed paramylon [1,3]. This difference is presumably due to the presence in Euglena of various other beneficial substances in addition to paramylon. The same tendency was also observed in the present study, as with the effects of Euglena were further enhanced by the co-ingestion of Euglena with vegetables. Based on these findings, enhancement of the effect of Euglena by its co-ingestion with vegetables was likely due to the presence of various additional beneficial constituents. Euglena is rich in vitamins and minerals as well as in many other nutrients [1]. Although these ingredients have a beneficial effect, they had not reached the amount that each ingredient exerts its effect in this study. Therefore, it was thought that the interaction of many ingredients is important. We previously showed that the diet of a Japanese person in 1975 was more beneficial for health maintenance than the diet of a modern day Japanese person [13,26,27,28]. The 1975 diet was characterized in one aspect by a diverse variety of food constituents and by the consumption of these constituents in small quantities. This led us to presume that consuming a variety of constituents in small amounts was very effective for health maintenance and therefore, co-consumption of Euglena with vegetables in the present study represented a positive enhancement of the diversity of food constituents.

In the present study, serum concentration of the inflammatory marker IL-6 and IL-1β [29] was reduced by Euglena intake and this reduction was enhanced by the co-consumption of Euglena with vegetables. This finding showed that vegetables enhanced the anti-inflammatory effect of Euglena [1,5]. Maintaining inflammatory markers at low levels prevents the onset of aging-associated diseases and is useful for health maintenance [29]. Thus, this finding suggested that the co-consumption of Euglena with vegetables is useful for achieving a long and healthy life. TBARS levels were reduced significantly by the co-consumption of Euglena with vegetables, indicating the possibility that this diet also reduced oxidative stress.

Adipocyte hypertrophy is the basis of lifestyle diseases such as diabetes mellitus, hyperlipidemia and arteriosclerosis [30]. Therefore, it is very important to maintain small adipocyte size. Histological examination of epididymal adipose tissue revealed the tendency of beneficial adipocyte-related changes in visceral fat weight. Although it is necessary to confirm our present results in animal models of obesity, it is possible that the co-consumption of Euglena with vegetables can prevent lifestyle diseases. Analysis of gene expression related to lipid metabolism in epididymal adipose tissue further confirmed the same trend: expression levels of *G6pdx* and *Srebf1* mRNAs encoding lipid accumulation-promoting molecules [31] were lower in Euglena + V group, whereas expression levels of *Pparg* [32], which promotes adipocyte growth and induces adipocyte size reduction and of *Hsl* [33], which induces lipolysis and adipocyte size reduction, exhibited the opposite tendency. The above findings showed that simultaneous intake of Euglena with vegetables enhanced the effects of Euglena.

The effect of the co-consumption of Euglena with vegetables on gut microbiota was examined to elucidate part of the mechanism of the anti-inflammatory effect of Euglena and its suppressive effect on visceral fat accumulation. The co-consumption of Euglena and vegetables caused a substantial increase in the following taxonomic groups: *Erysipelotrichaceae; Other*, *YS2;f__;g__* and *Lactobacillus*. The fraction of the bacterial subclass *Erysipelotrichaceae; Other* has been shown to increase following the consumption of β-glucans and inulin [34]. These bacteria participate in the production of short-chain fatty acids [35]. In the present study, the fraction of *Erysipelotrichaceae; Other* was increased by the consumption of vegetables rich in soluble dietary fiber, which also caused an actual increase in short-chain fatty acid concentrations. The beneficial effects of short-chain fatty acids include the anti-inflammatory action due to strengthened intestinal tract barrier function and anti-obesity effect due to increased energy consumption through GPR41 and GPR43 in the liver [36,37,38]. In this study, the co-consumption of Euglena with vegetables was associated with higher serum concentrations of short chain fatty acids and lower visceral fat accumulation. These findings suggest that the effects of vegetables observed in this study may be attributed to enhanced production of short-chain fatty acids. *YS2;f__;g__* bacteria were shown to promote the biosynthesis of vitamins B and K [39,40]. It is possible that ingestion of vegetables led to greater uptake of vitamins with beneficial effects for the body due to the associated increase in *YS2;f__;g__* amount. Lactobacillus produces lactic acid by sugar metabolism in the intestines and promotes the production of GABA and other useful substances [41,42]. Lactic acid suppresses the growth of harmful bacteria susceptible to acidic conditions and exerts an anti-inflammatory effect. We confirmed that the co-consumption of Euglena with vegetables resulted in elevated levels of GABA, a substance with a stress-reducing effect [16,43]. It is possible that the increase in Lactobacillus observed in this study was one of important effects of vegetable consumption. Furthermore, the co-consumption of Euglena with vegetables caused a substantial reduction in the genera *Staphylococcus*, *Jeotgalicoccus*, *Sporosarcina*, *Streptococcus*, *Sutterella* as well as in the representatives of the family *Clostridiaceae; Other*. *Staphylococcus aureus* causes a variety of diseases and belongs to the genus *Staphylococcus* [44]. In the present study, compared to the fraction of *Staphylococcus* in control group, the representatives of this genus were more abundant in mice that consumed paramylon but less abundant in mice that consumed Euglena and even less frequent after the co-consumption of Euglena with vegetables. The fractions of *Jeotgalicoccus* and *Sporosarcina* in the gut microbiota were shown to increase during hepatitis induced by artificial sweeteners [45]. An increase in the number of microorganisms of these genera may therefore promote inflammation. In the present study, differences in the fractions of these genera between experimental groups exhibited similar pattern to that of *Staphylococcus*: their levels were higher after paramylon ingestion, lower after Euglena ingestion and decreased even further after the co-consumption of Euglena with vegetables. *Streptococcus* causes streptococcal infection [46]. Therefore, an increase in the abundance of this genus is also presumed to promote inflammation. In the present study, changes in *Streptococcus* levels were similar to those in *Staphylococcus*: *Streptococcus* levels were (relative to control group) higher after paramylon consumption but lower in animals that received Euglena in the diet and minimal after the co-consumption of Euglena with vegetables. Based on the changes observed in these four genera, the co-consumption of Euglena with vegetables likely protected against the negative effects of paramylon. *Sutterella* fraction was shown to correlate positively with intestinal inflammation [47]. Furthermore, the family *Clostridiaceae; Other* includes harmful bacteria that cause food poisoning and other conditions [48]. In the present study, the abundance values of these taxa after feeding mice with paramylon, Euglena and Euglena + V vegetables were progressively lower than in control group. These findings suggest that the co-consumption of Euglena with vegetables enhanced the positive effects of paramylon. In particular, the abundance of beneficial bacteria was higher, whereas the fractions of harmful bacteria were lower in mice that consumed Euglena with vegetables, which may be one of the mechanisms of the positive effect of vegetables.

In this study, no significant effect of paramylon intake was observed compared to the parameters measured after control diet intake. The apparent lack of effect could be due to the fact that the dose of paramylon in this study was small compared to those used in previous studies. It is likely that increasing paramylon intake would make its effect more pronounced, because the tendencies of lower inflammatory marker concentration and suppressed visceral fat accumulation were observed following paramylon intake. Therefore, studies similar to ours in which paramylon effects are shown more efficiently, will be very important.

## 5. Conclusions

The above findings showed that the co-consumption of Euglena with vegetables enhanced its anti-inflammatory effect and suppressive effect on visceral fat accumulation and suggested that modulation of gut microbiota could be one of the mechanisms of these changes. It is therefore extremely important that more effective methods of consuming Euglena-based products are determined. In future, we would like to establish more effective methods of introducing Euglena and to examine potential positive effects of Euglena and vegetable co-consumption in animal models of obesity and verify these effects in humans.

## Figures and Tables

**Table 1:** Composition of test diets.

	Control Diet	Paramylon Diet	Euglena Diet	Euglena + Vegetables Diet
Ingredient	(g/100 g diet)			
Casein	16.3	16.3	16.1	16.1
DL-Methionine	0.3	0.3	0.3	0.3
Corn Starch	33.8	33.7	33.5	33.3
Maltodextrin 10	8.4	8.4	8.3	8.3
Sucrose	28.5	28.4	28.2	28.1
Cellulose	4.2	4.2	4.2	4.1
Corn Oil	4.4	4.4	4.4	4.3
Mineral Mix (S10001)	2.9	2.9	2.9	2.9
Calcium Carbonate	0.3	0.3	0.3	0.3
Vitamin Mix (V10001)	0.8	0.8	0.8	0.8
Choline Bitartrate	0.2	0.2	0.2	0.2
Paramylon	-	0.3	-	-
Euglena	-	-	1.0	1.0
Barley leaf	-	-	-	0.1
Kale	-	-	-	0.1
Aghitaba	-	-	-	0.1
Others				0.1
	(g/100 g diet)			
Total Dietary Fiber	4.2	4.5	4.5	4.7
	(energy %)			
Protein	17.1	17.1	17.3	17.5
Carbohydrate	72.7	72.7	72.2	72.1
Fat	4.5	4.5	4.7	4.6
Energy (kcal/100 g)	389	388	388	388

Control diet, normal diet; Paramylon diet, the normal diet containing 0.3% Paramylon; Euglena diet, the normal diet containing 1.0% Euglena; Euglena + Vegetables diet, the normal diet containing 1.0% Euglena and 0.3% Vegetables.

**Table 2:** Primer pairs used for the real time qRT-PCR analysis.

Genbank ID	Gene Name		Primer Sequence (5′ to 3′)
NM_007988	*Fasn*	Forward	CCTGGATAGCATTCCGAACCTG
		Reverse	TTCACAGCCTGGGGTCATCTTTGC
NM_008062	*G6pdx*	Forward	TGGGTCCACCACTGCCACTTTTG
		Reverse	ATTGGGCTGCACACGGATGACCA
NM_001039507	*Hsl*	Forward	TTCTCCAAAGCACCTAGCCAA
		Reverse	TGTGGAAAACTAAGGGCTTGTTG
M29546	*Me*	Forward	GAAAGAGGTGTTTGCCCATGA
		Reverse	AATTGCAGCAACTCCTATGAGG
NM_011146	*Pparɤ*	Forward	GGAAGACCACTCGCATTCCTT
		Reverse	TCGCACTTTGGTATTCTTGGAG
NM_011480	*Srebf1*	Forward	GGAGACATCGCAAACAAGC
		Reverse	TGAGGTTCCAAAGCAGACTG
NM_007393	*Actb*	Forward	GGCTGTATTCCCCTCCATCG
		Reverse	CCAGTTGGTAACAATGCCATGT

*Fasn*, fatty acid synthase; *G6pdx*, glucose-6-phosphate 1-dehydrogenase X; *Hsl*, hormone sensitive lipase; *Me*, malic enzyme; *Pparɤ*, peroxisome proliferator-activated receptor gamma; *Srebf1*, sterol regulatory element-binding protein 1c; *Actb*, actin beta.

**Table 3:** Body weights, food intake and tissue weights.

	CO	Paramylon	Euglena	Euglena + V
Initial body weight (g)	22.9 ± 0.3	22.9 ± 0.3	22.9 ± 0.3	22.9 ± 0.3
Final body weight (g)	29.4 ± 0.6	28.5 ± 0.4	28.3 ± 0.5	28.0 ± 0.9
Food intake (g/day)	3.01 ± 0.04	2.99 ± 0.05	3.09 ± 0.06	2.96 ± 0.06
Energy intake (kcal/day)	11.8 ± 0.2	11.7 ± 0.2	12.1 ± 0.2	11.5 ± 0.2
Tissue weight (g/100 g body weight)				
Brain	1.57 ± 0.04	1.59 ± 0.04	1.59 ± 0.03	1.58 ± 0.04
Heart	0.45 ± 0.01	0.45 ± 0.01	0.46 ± 0.01	0.44 ± 0.01
Lung	0.76 ± 0.05	0.87 ± 0.08	0.89 ± 0.11	0.75 ± 0.05
Liver	3.31 ± 0.03	3.41 ± 0.03	3.39 ± 0.06	3.37 ± 0.04
Pancreas	0.68 ± 0.03	0.78 ± 0.09	0.65 ± 0.03	0.73 ± 0.03
Spleen	0.29 ± 0.02	0.28 ± 0.01	0.26 ± 0.01	0.28 ± 0.01
Kidney	1.10 ± 0.03	1.18 ± 0.03	1.14 ± 0.04	1.10 ± 0.04
White adipose tissue				
Mesenteric	1.29 ± 0.05	1.22 ± 0.12	0.90 ± 0.12	0.91 ± 0.12
Perinephric	1.38 ± 0.10 ^a^	1.09 ± 0.16 ^ab^	0.70 ± 0.07 ^bc^	0.65 ± 0.08 ^c^
Epididymal	2.61 ± 0.15 ^a^	2.40 ± 0.26 ^a^	1.55 ± 0.14 ^b^	1.51 ± 0.16 ^b^
Total	5.28 ± 0.28 ^a^	4.70 ± 0.47 ^a^	3.15 ± 0.30 ^b^	3.01 ± 0.30 ^b^

Values are mean ± SE, *n* = 10. Different superscript letters indicate significantly different means at *p* < 0.05 (refer to “Result” in detail). CO, a group fed the control diet; Paramylon, a group fed the normal diet containing 0.3% Paramylon; Euglena, a group fed the normal diet containing 1.0% Euglena; Euglena + V, a group fed the normal diet containing 1.0% Euglena and 0.3% Vegetables.

**Table 4:** Biochemical parameter in serum and liver.

	CO	Paramylon	Euglena	Euglena + V
**Serum**				
TG (mmol/L)	1.38 ± 0.08	1.39 ± 0.07	1.19 ± 0.07	1.23 ± 0.08
TC (mmol/L)	3.29 ± 0.15	3.64 ± 0.12	3.28 ± 0.12	3.59 ± 0.07
PL (mmol/L)	73.9 ± 2.7	79.9 ± 3.1	77.5 ± 2.2	82.7 ± 1.5
Glucose (mmol/L)	5.55 ± 0.27	5.61 ± 0.16	4.88 ± 0.18	5.41 ± 0.27
Insulin (μg/L)	0.19 ± 0.01	0.18 ± 0.01	0.16 ± 0.01	0.16 ± 0.01
TBARS (µmol/L)	7.16 ± 0.33 ^ab^	7.79 ± 0.38 ^b^	7.44 ± 0.31 ^ab^	6.35 ± 0.35 ^a^
ALT (IU/L)	5.03 ± 0.41	4.63 ± 0.29	4.48 ± 0.37	4.09 ± 0.21
AST (IU/L)	54.6 ± 3.3	60.5 ± 4.4	46.7 ± 4.6	54.9 ± 4.1
Leptin (ng/mL)	4.27 ± 0.80	3.96 ± 0.69	3.38 ± 0.83	2.24 ± 0.47
**Liver**				
TG (μmol/g)	43.2 ± 7.1	41.9 ± 5.4	42.9 ± 4.3	39.6 ± 4.1
TC (μmol/g)	8.59 ± 1.17	8.17 ± 1.18	7.60 ± 1.17	7.13 ± 0.44
PL (μmol/g)	35.2 ± 0.6	35.1 ± 1.0	34.7 ± 0.7	33.4 ± 1.0
TBARS (nmol/g)	73.1 ± 2.7 ^b^	68.1 ± 2.7 ^ab^	65.6 ± 3.1 ^ab^	60.2 ± 2.8 ^a^

Values are mean ± SE, *n* = 10. Different superscript letters indicate significantly different means at *p* < 0.05 (refer to “Result” in detail). CO, a group fed the control diet; Paramylon, a group fed the normal diet containing 0.3% Paramylon; Euglena, a group fed the normal diet containing 1.0% Euglena; Euglena + V, a group fed the normal diet containing 1.0% Euglena and 0.3% Vegetables.

**Table 5:** Gut microbiota (genus level) that increased by more than 2 times or decreased to 1/2 or less in the Euglena + V group compared to the Euglena group.

	CO	Paramylon	Euglena	Euglena + V	Euglena + V/Euglena
*Erysipelotrichaceae; Other* (%)	0.00	0.01	0.01	0.04	4.01
*YS2;f__;g__* (%)	0.01	0.02	0.03	0.05	2.10
*Lactobacillus* (%)	2.51	2.08	2.41	4.91	2.04
*Staphylococcus* (%)	0.02	0.18	0.09	0.03	0.28
*Jeotgalicoccus* (%)	0.07	0.66	0.10	0.02	0.21
*Sporosarcina* (%)	0.01	0.90	0.14	0.02	0.14
*Streptococcus* (%)	0.00	0.02	0.01	0.00	0.10
*Sutterella* (%)	0.15	0.02	0.01	0.00	0.10
*Clostridiaceae; Other* (%)	1.01	0.01	0.00	0.00	0.01

**Table 6:** mRNA expression level in white adipose tissue (ratio).

	CO	Paramylon	Euglena	Euglena + V	Gene Function
*Fasn*	1.00 ± 0.10	1.02 ± 0.14	0.95 ± 0.06	0.82 ± 0.07	Fatty acid synthesis
*G6pdx*	1.00 ± 0.14 ^a^	0.79 ± 0.08 ^ab^	0.74 ± 0.10 ^ab^	0.53 ± 0.09 ^b^	
*Me*	1.00 ± 0.06 ^ab^	1.27 ± 0.16 ^a^	0.97 ± 0.06 ^ab^	0.81 ± 0.07 ^b^	
*Srebf1*	1.00 ± 0.11 ^ab^	1.09 ± 0.11 ^a^	0.77 ± 0.03 ^ab^	0.76 ± 0.05 ^b^	
*Pparɤ*	1.00 ± 0.06 ^b^	1.06 ± 0.06 ^b^	1.13 ± 0.08 ^ab^	1.36 ± 0.08 ^a^	Cell division
*Hsl*	1.00 ± 0.06 ^c^	1.09 ± 0.08 ^bc^	1.40 ± 0.13 ^ab^	1.50 ± 0.08 ^a^	Lipolysis

Values are mean ± SE, *n* = 10. Different superscript letters indicate significantly different means at *p* < 0.05 (refer to “Result” in detail). CO, a group fed the control diet; Paramylon, a group fed the normal diet containing 0.3% Paramylon; Euglena, a group fed the normal diet containing 1.0% Euglena; Euglena + V, a group fed the normal diet containing 1.0% Euglena and 0.3% Vegetables.

## Founding

This research received no external funding.
